# Elucidating discrepancies among reported wildlife trade: A response to “Is biomedical research demand driving a monkey business?” by Warne et al. (2023)

**DOI:** 10.1016/j.onehlt.2024.100686

**Published:** 2024-01-26

**Authors:** Jonathan E. Kolby, Jamie K. Reaser

**Affiliations:** Smithsonian's National Zoo & Conservation Biology Institute, Front Royal, VA 22630, USA

**Keywords:** Wildlife trade, CITES, LEMIS, Conservation, Public health, Zoonotic

## Abstract

Accurate representation of international trade in live macaques is a matter of considerable conservation and public health relevance. Our response to Warne et al. (2023) describes data handling and interpretation deficits among evaluation of non-human primate trade and the treatment of international wildlife trade more generally. In contradiction to statements made by Warne and colleagues, according to US trade records, thousands of live macaques have been exported to the United States from China since 2018 and this high-risk trade in endangered species continues to warrant attention from conservation and public health perspectives. The accuracy of wildlife trade data used to influence policy decisions also warrants greater scrutiny by researchers and decision-makers applying their results.

As a tenet of scientific integrity, the public must be able to trust the science and scientific process informing public policy decisions. In the international wildlife trade context, scientific findings influence policy decisions across multiple regulatory frameworks, including those intended to protect threatened and endangered species, mitigate invasive species and/or zoonotic pathogen spillover risk, and safeguard animal welfare. Our response to Warne et al. [1] offers an opportunity to address data handling and interpretation shortfalls in the non-human primate trade context and serves as a cautionary note for the handling of international wildlife trade data more broadly. The majority of traded animals discussed by the authors are long-tailed macaques (*Macaca fascicularis*), a species recently uplisted from Vulnerable to Endangered by the IUCN Red List of Threatened Species [2], largely due to the increasing threat posed by international trade demand. Non-human primates serve as vectors of zoonotic pathogens with severe public health consequences, such as Ebola virus [3], and precise characterization of trade dynamics along the supply chain, from origin to destination, is necessary to mitigate spillover risks.

Warne et al. [1] examined the international trade in live macaques (*Macaca* spp.), reporting that the majority of live macaques exported from Asia are destined for the United States. The authors state that, until Chinese exports of live macaques were halted in 2019, China was the main supplier of these non-human primates to United States and that Cambodia then emerged as the new primary global supplier. We reviewed the data provided by the authors, as well as the original set of trade records maintained in the CITES Trade Database [4] from which their conclusions were drawn. Our analyses identify macaque trade activity that contradicts the observations and conclusions by Warne and colleagues. We are aware that data handling and interpretation errors are not unusual for these datasets due to inherent data capture complexities and data reporting irregularities. Yet, we recognize that corrections are now warranted as the publication of misinformation arising from these information sources can be highly consequential for national and international policy making.

Warne et al. [1] state: “Of particular interest were the trade discrepancies reported between 2019 and 2020, wherein Cambodia significantly increased their exports of macaques whilst China, traditionally one of the largest suppliers of macaques, ceased all exports.” In stark contrast to this conclusion, our analyses of CITES Trade Database records indicates that during 2019, the United States recorded and reported a substantial quantity of live macaques imported from China. This import trade involved 15,204 long-tailed macaques (*Macaca fascicularis*) and 972 rhesus monkeys (*Macaca mulatta*). The error made by Warne and colleagues arose because they: 1) only considered CITES Trade Database records submitted by the exporting country (China), 2) missed certain records of export that China did submit, and 3) chose to exclude from consideration legitimate import records submitted by destination countries (i.e., the United States, Japan, and South Korea) [4].

It appears the authors have conflated their perceived absence of reported trade with the absence of actual wildlife trade, an understandable misinterpretation of CITES wildlife trade data. The physical arrival of live macaques officially reported to the CITES Secretariat by the United States, Japan, and South Korea should be considered legitimate trade records, even if corresponding records of exportation by China are unavailable. Had the authors investigated the numbers provided by both the exporting and importing countries, it would have been readily apparent that China was still exporting live macaques to the United States. China's export data were submitted to the CITES Secretariat on 2 February 2021 [6] and were uploaded into the CITES Trade Database in March 2022. These data indicate the export of 708 (360 *M. fascicularis* and 348 *M. mulatta*) live macaques to the United States in 2019. As the methods described by Warne et al. (2023) stated that CITES trade data were queried on 6 October 2022, these export records were present in the database during their study period. Although Cambodia's reported trade in live macaques did rise considerably around this same time, as expressed by the authors, the export trade in China continued and must be acknowledged.

The striking difference between the numbers of traded live macaques reported by the United States in 2019 compared to those reported by China warrants investigation by the US Fish and Wildlife Service's (USFWS) Office of Law Enforcement (OLE) and the CITES Secretariat. Although China did report that some of these animals were exported to the United States during 2019, this only accounted for approximately 5% of the quantity recorded by the United States upon arrival [4]. The validity of CITES export documents that accompanied the additional 14,844 long-tailed macaques (*M. fascicularis*) and 624 rhesus monkeys (*M. mulatta*) in excess of their reported exports should also be explored by USFWS OLE to ensure these animals were legally imported. A pattern of underreporting of live macaque exports by China is not unique among animals sent to the United States; both Japan and South Korea also reported imports of live macaques from China in 2019 for which the CITES Trade Database shows no export records. Although these types of multinational wildlife trade data discrepancies can sometimes be explained by staggered or delayed dates of report submission, it should be noted that all countries named herein submitted their 2019 CITES wildlife trade records no later than September 2022 [6].

The accurate conclusion that should be drawn from the CITES Trade Database macaque data is that *thousands of live macaques have been exported to the United States from China since 2018 and this high-risk trade in endangered species continues to warrant attention from conservation and public health perspectives*.

Still, further caution is warranted in data handling and interpretation. CITES trade data provided by the United States is derived from the USFWS OLE database known as LEMIS (Law Enforcement Management Information System). Errors can occur when data are entered into LEMIS, as well as when LEMIS data are extracted and prepared for the CITES Annual Report. As part of a Congressionally directed project aimed at zoonotic disease risk mitigation, we are currently reviewing LEMIS data in detail, including for non-human primates. Although still in the initial stages of this process, we have identified substantial errors in macaque data input, including for the years covered in the study by Warne and colleagues [1]. It is clear that greater scrutiny of data submitted in CITES Annual Reports is required.

Regarding the authors' characterization of Cambodia's trade in macaques, Warner et al. [1] failed to acknowledge that, as of 2 June 2023, Cambodia had not yet submitted their 2018 CITES Annual Report data. The deadline for submission of a CITES Annual Report is 31 October of the year following the year in which trade occurred. However, it is not uncommon for reports to be submitted in a highly staggered fashion, sometimes years later than required. This results in inconsistent and staggered updating of the CITES Trade Database. Interpretation challenges and limitations caused by the non-synchronized submission and availability of international wildlife trade data affect virtually all trade studies that draw conclusions from the CITES Trade Database. To strengthen the scientific integrity of wildlife trade summaries based on these data, publications of results should include careful terminology that expresses distinction between the absence of wildlife trade versus the absence of wildlife trade data for actual trade events.

In the absence of Cambodian export data for 2018, wildlife trade data can be reviewed for those countries that are known to import macaques. These records indicate that at least 9610 live macaques were exported from Cambodia in 2018, of which 150 animals were bred in captivity in China and the remainder in Cambodia. Because a query of Cambodia's export data in the CITES Trade Database doesn't produce the requisite data [4], the authors mistakenly describe the Cambodian exports as "indirect trade," suggesting that the macaques sourced from Cambodia were re-exported through an intermediary country before reaching their destination. In actuality, direct trade took place between Cambodia and the importers. The accurate depiction of trade routes is especially important when describing transport patterns of animals capable of carrying dangerous zoonotic pathogens.

Despite the absence of Cambodia's 2018 CITES Annual Report, Cambodia has submitted more recent trade reports (2019–2021) with data that currently populates the CITES Trade Database. It should thus be acknowledged that Cambodia's 2018 trade data may be submitted at any time and analysis of these records will unpredictably impact the conclusions drawn by Warne and colleagues.

The CITES Secretariat is well positioned to proactively urge caution in the analysis, interpretation, and application of the publicly available records maintained in the less-than-perfect CITES Trade Database, as it is intended as a regulatory oversight tool rather than a dataset curated to meet standards of scientific rigor. The CITES Trade Database website provides a document called, "A guide to using the CITES Trade Database" that explicitly warns data users that discrepancies may arise between importer and exporter reported data for eight different reasons, primarily attributed to differences in how trade is described and reported [5]. We also call attention to three additional opportunities for discrepancies to emerge: a) the mishandling of records in national datasets prior to their submission in CITES Annual Reports, b) mistakes made during the subsequent transfer of these records from the CITES Annual Reports into the CITES Trade Database, and/or c) when exports are accompanied by fraudulent CITES permits. Because CITES trade data will continue to feed into scientific studies, many intended to influence domestic and international trade policies, they should be presented together with careful narratives and caveats reflecting the inherent complexity and limitations of the reported information. When informing policy decisions likely to have substantial consequence, we strongly encourage the scientific community to assess national wildlife trade datasets for accuracy rather than simply relying on CITES derived data.

## CRediT authorship contribution statement

**Jonathan E. Kolby:** Writing – review & editing, Writing – original draft, Methodology, Investigation, Conceptualization. **Jamie K. Reaser:** Writing – review & editing, Supervision, Funding acquisition, Conceptualization.

## Declaration of competing interest

All authors declare that we have no conflicts of interest.

## Data Availability

No novel data were created for the preparation of this manuscript.
